# Immunohistological analysis reveals IgG1-dominant immunophenotype of tubulointerstitial nephritis unassociated with IgG4-related diseases

**DOI:** 10.1007/s11255-024-03966-1

**Published:** 2024-02-21

**Authors:** Toshiki Hyodo, Shigeo Hara, Shunsuke Goto, Hideki Fujii, Shinichi Nishi, Tomoko Horinouchi, Kandai Nozu, Norishige Yoshikawa, Akihiro Yoshimoto, Tomoo Itoh

**Affiliations:** 1 Department of Diagnostic Pathology, Kobe University Graduate School of Medicine, Kobe, Japan; 2 Division of Nephrology and Kidney Center, Kobe University Graduate School of Medicine, Kobe, Japan; 3 Department of Pediatrics, Kobe University Graduate School of Medicine, Kobe, Japan; 4 Clinical Research Center, Takatsuki General Hospital, Takatsuki, Japan; 5 Department of Nephrology, Kobe City Medical Center General Hospital, Kobe, Japan; 6 Department of Diagnostic Pathology, Kobe City Medical Center General Hospital, Kobe, Japan

**Keywords:** IgG subclass, IgG1, IgG4, Plasma cells, Tubulointerstitial nephritis

## Abstract

**Purpose:**

Tubulointerstitial nephritis (TIN) has various etiologies, including IgG4-related disease (IgG4-RD), autoimmune diseases, antineutrophil cytoplasmic antibody (ANCA)-associated vasculitis (AAV), and others. IgG4-positive plasma cell infiltration can occasionally be found in TIN unrelated to IgG4-RD. Therefore, there may be problems with usage of IgG4 immunostaining to differentiate between TIN with and TIN without IgG4-RD. This study aimed to compare the proportion of plasma cells that are positive for each IgG subclass and to clarify the predominant IgG subclass trends and clinical characteristics associated with IgG4-RD and non-IgG4-related interstitial nephritis.

**Methods:**

The study enrolled 44 cases of TIN: 6 of IgG4-RD, 8 of autoimmune disease, 9 of AAV, and 21 of unknown disease group. In addition to clinical characteristics, IgG subclass composition of interstitial plasma cells was evaluated among 4 groups by immunohistochemistry.

**Results:**

IgG1 was the predominant IgG subclass in TIN unrelated to IgG4-RD. In the IgG4-RD group, the IgG subclass rate was high in both IgG1 and IgG4. The rate of average IgG4-positive cells was significantly lower in the autoimmune disease group and unknown disease group compared with the IgG4-RD group.

**Conclusion:**

The present study revealed IgG1-dominant immune profiles of TIN unrelated to IgG4-RD. Further investigation is required to elucidate the clinicopathological differences between IgG1-dominant and IgG4-dominant groups in IgG4-RD.

**Supplementary Information:**

The online version contains supplementary material available at 10.1007/s11255-024-03966-1.

## Introduction

Tubulointerstitial nephritis (TIN) can be one of the rare causes of kidney injury. Its etiology is highly heterogeneous, but clinically it can be broadly divided into acute and chronic forms. Pathologically, neutrophils and eosinophils may predominate in acute TIN, while lymphocytes and plasma cells are common in both acute and chronic TIN [1]. The prototypical example of TIN with prominent plasma cell infiltrations is IgG4-related disease (IgG4-RD). Representative hallmarks include numerous infiltrates of IgG4-positive plasma cells and characteristic sclerotic fibrosis involving multiple organs. The comprehensive diagnostic criteria for IgG4-RD are defined as a serum IgG4 level > 135 mg/dl, histologically an IgG4/IgG plasma cell ratio of > 40%, and IgG4-positive cell counts of > 10 in a high magnification field [2, 3]. Meanwhile, diagnosis of IgG4-RD is sometimes complicated by atypical cases, such as IgG4-RD with normal serum IgG4 levels, non-IgG4-RD with high serum IgG4 levels [4], or non-IgG4-related interstitial nephritis with significant IgG4-positive plasma cell infiltration [5, 6]. Diagnostic criteria of IgG4-RD may in fact be fulfilled by various diseases with hypergammaglobulinemia, including antineutrophil cytoplasmic antibody (ANCA)-associated vasculitis (AAV), autoimmune diseases such as systemic lupus erythematosus (SLE), and multicentric Castleman’s disease. This could be a potential cause of diagnostic pitfalls [4, 7].

IgG subclass analysis has been widely used to differentiate the cause of immune-complex glomerulonephritis, especially membranous nephropathy (MN), which can be classified into IgG4-dominant/co-dominant phenotype in primary MN and non-IgG4-dominant/co-dominant phenotype in secondary MN [8]. Meanwhile, IgG subclass composition of interstitial plasma cells infiltrating into tissues has not been widely investigated in the kidneys [9]. In this study, we examine the IgG subclass composition of plasma cells in various forms of TIN. Our goal is to compare the proportion of plasma cells that are positive for each IgG subclass and to clarify the predominant IgG subclass trends and clinical characteristics associated with IgG4-RD and non-IgG4-related interstitial nephritis.

## Materials and methods

### Patients

The present study enrolled pathologically diagnosed cases of TIN (IgG4-RD, autoimmune diseases, AAV, and others/unknown) from Kobe University Hospital and Kobe City Medical Center General Hospital between January 1996 and December 2021. Clinical information at the time of diagnosis was extracted from medical records for the following: age, sex, medications, the period from disease onset to the biopsy, levels of serum IgG, IgG4, complement component 3 (C3), creatinine (Cr), estimated glomerular filtration rate (e-GFR), urinary *N*-acetyl-beta-glucosaminidase (NAG), urinary beta-2 microglobulin (β2MG), hematuria, and proteinuria. This study was approved by the ethics committees of Kobe University Hospital (B210181) and Kobe City Medical Center General Hospital (21264).

### Immunohistological studies

IgG1, IgG2, IgG3, and IgG4-positive plasma cells were evaluated by the immunohistochemical method, using an automatic immunohistochemical staining experiment on BOND™ MAX (Leica Biosystems, Wetzlar, Germany). Paraffin sections were cut to 4 μm thickness, deparaffinized, and stained with rabbit anti-human IgG1 (1:10,000; ab201485), anti-IgG2 (1:10,000; ab134050), and anti-human IgG3 (1:1000; ab193172), from Abcam plc., Cambridge, UK, and Mouse anti-human IgG4 (1:500; A-10651), from Invitrogen/Thermo Fisher Scientific, Waltham, MA, USA. Two pathologists (TH and SH) measured IgG1, IgG2, IgG3, and IgG4-positive plasma cells in each of the three fields with high magnification (× 400). The percentage of IgG1, IgG2, IgG3, and IgG4-positive plasma cells was determined by counting the number of positive cells, and the mean percentage was defined as *IgG subclass rate*. Cases were excluded if < 10 plasma cells were observed in each field.

### Statistical analysis

Statistical analysis was performed using EZR Software (Jichi Medical University, Japan, http://www.jichi.ac.jp/saitama-sct/). If the IgG subclass rate was significantly different between IgG4-RD and the other three groups, the post hoc test confirmed significant differences between the three diseases. *p* values reported in this paper were corrected for multiple testing and were considered significant when their corresponding *p* value was less than 0.05 (*p* < 0.05).

## Results

The total renal biopsy number was 4367 (2750 cases in Kobe University and 1617 cases in Kobe City Medical Center General Hospital, respectively) in this study. Of 64 consecutive cases of pathologically confirmed TIN, five cases with < 10 infiltrating plasma cells and 15 various causing diseases [glomerulonephritis other than AAV (*n* = 3), drugs (*n* = 3), infection (*n* = 2), tubulointerstitial nephritis and uveitis syndrome (*n* = 2), Castleman disease/sarcoidosis/Still’s disease/ulcerative colitis/angioimmunoblastic T-cell lymphoma (*n* = 1)] were excluded. The clinical characteristics for each disease group are shown in Table 1. The total number of eligible cases thus amounted to 44, out of which 41 cases were adult (male-to-female ratio, 21:20; average age, 60.1 years [36–81]) and the remaining three were pediatric cases (male-to-female ratio 3:0, average age, 14 years [13–15]). The study cohort consisted of six cases of IgG4-RD, eight cases of autoimmune diseases [five cases of Sjogren’s syndrome, two cases of primary biliary cholangitis (PBC), and one case of SLE], nine cases of AAV (two cases of PR3-ANCA positive AAV and seven cases of MPO-ANCA positive AAV), and 21 cases of unknown disease group. Average age was highest in the IgG4-RD group. Male patients were predominant in IgG4-RD and in the unknown disease group. The period from disease onset to the biopsy was highest in the autoimmune disease group (44.3 months). By definition, serum IgG4 level was the highest (1103 mg/dl) in the IgG4-RD group. Renal function was the worst in the unknown disease group, with serum Cr 3.76 mg/dl. In the AAV group, all cases were positive for both hematuria and proteinuria. Urinary β2MG was highest in the unknown disease group, followed by autoimmune disease group. Urinary NAG was elevated in all groups. Detailed clinical characteristics of all cases are shown in Supplementary Table 1.

**Table 1:** Clinical information by variable causes of all cases

	IgG4-RD	Autoimmune disease	AAV	Unknown
Number of patients	6	8	9	21
Age	67.2 (50–81; 68.5)	47.1 (14–70; 47.5)	61.7 (46–76; 62)	55.8 (13–78; 62)
Male (%)	5 (83.3)	2 (25.0)	4 (44.4)	13 (61.9)
Period (month)^1^	9.33 (2 m–2 y; 6 m)	44.30 (2.5 m–14 y; 2 y) (*n*=5)	1.86 (0.25 m–3 m; 1.5 m)	9.47 (0.25 m–9 y; 2 m) (*n*=20)
Causes	IgG4-RD	Sjogren’s syndrome (*n*=5), PBC (*n*=2), SLE (*n*=1)	MPO (*n*=7), PR3 (*n*=2)	Unknown
IgG (mg/dl) [861–1747]	3505 (2048–4936; 3447)	2600 (1016–6352; 2060) (*n*=7)	1361 (480–2291; 1246)	1650 (383–2647; 1670)
IgG4 (mg/dl) [5–117]	1103 (288–3120; 788.5)	27.7 (12.6–63.5; 17.2) (*n*=5)	NA	167.3 (7.4–1090; 36.5) (*n*=8)
Cr (mg/dl) [0.65–1.07]	1.26 (0.92–1.54; 1.26)	1.20 (0.65-1.75; 1.22) (*n*=7)	2.20 (0.66–4.41; 1.34)	3.76 (1.07–16.94; 2.23)
e-GFR (ml/min/1.73 m^2^)	45.8 (37–65; 44.0)	43.9 (29–79.7; 40) (*n*=7)	36.4 (8.2–72.7; 32)	21.4 (2–42.2; 21.6)
C3 (mg/dl) [73~138]	60.8 (40–105; 50) (*n*=5)	87 (24–117; 100) (*n*=6)	111.9 (82–126; 121.5) (*n*=8)	115.3 (79–167; 111) (*n*=20)
Hematuria	(3/1/1/1/0)	(3/1/2/1/0) (*n*=7)	(0/0/4/2/3)	(5/4/6/2/4)
(−/±/1+/2+/3+)				
Proteinuria	(1/2/2/0/1/0)	(1/3/1/2/0/0) (*n*=7)	(0/0/0/2/7/0)	(3/2/11/3/1/1)
(−/±/1+/2+/3+/4+)				
β2MG (μg/L) [≤ 289]	9428.2 (40–399667; 4164.5)	21191.8(6287–43060; 19838) (*n*=6)	4804.7(140–23641; 1383)	28400.3(193–96070; 19254) (*n*=20)
NAG (IU/L) [≤ 5.7]	18.37 (2.9–51.5; 10.05)	9.69 (3.6–30.7; 5.4) (*n*=7)	17.00 (0.27–31.7; 17.7)	17.05 (2.1–43.2; 14.4) (*n*=19)

All data were expressed as mean value (minimum–maximum; median value). Some clinical data of several cases were unavailable.

Period^1^ indicates the time from patient’s symptom until biopsy （m, month; y, year）. Clinical information of the time between disease onset and diagnosis was unavailable in some cases.

**Fig. 1:**
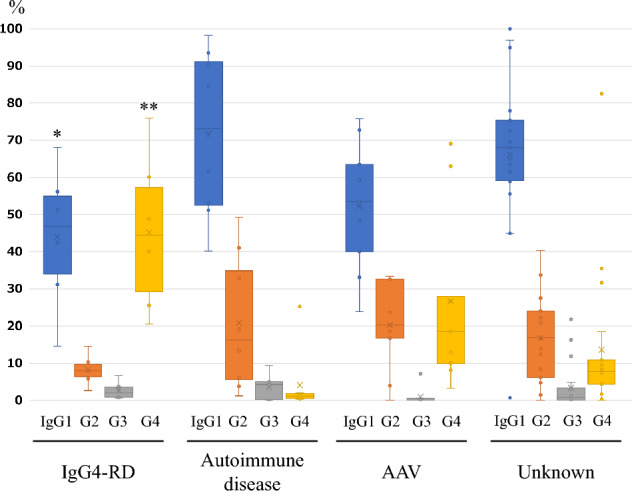
IgG subclass profiles of four TIN groups. The average IgG subclasses percentage of four TIN groups classified by etiologies (IgG4-RD, autoimmune disease, AAV, and others) is shown in Box-and-Whisker plot. Significant p values of IgG subclass between IgG4RD and other 3 groups are indicated by * (IgG1, *p* = 0.036) and ** (IgG4, *p* < 0.01), respectively. There were no significant differences in the percentages of IgG2 (*p* = 0.344) or IgG3 (*p *= 0.115) positive plasma cells between IgG4-RD and the other groups. Blue, orange, gray, and yellow boxes indicate IgG1, IgG2, IgG3, and IgG4, respectively.

**Fig. 2:**
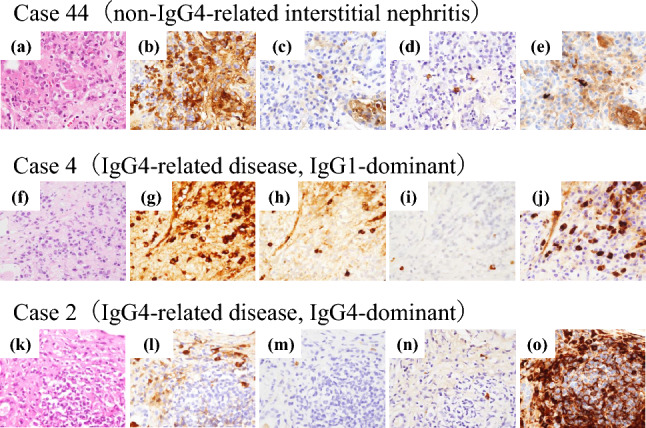
Images of IgG subclass immunohistochemistry. Representative images of non-IgG4-related interstitial nephritis (**a–e**, case 44; etiology unknown), IgG1-dominant IgG4-RD (**f–j**, case 4), and IgG4-dominant IgG4-RD (**k–o**, case 2).** b–e, g–j**, and **l–o** are the images of IgG1 (**b, g, l**), IgG2 (**c, h, m**), IgG3 (**d, i, n**), and IgG4 (**e, j, o**), respectively. In a non-IgG4-related interstitial nephritis (case 44; etiology unknown), IgG1-positive plasma cells were most abundant. IgG4-RD cases were represented by IgG1-dominant (case 4) and IgG4-dominant (case 2) subgroups.

*AAV* ANCA-associated vasculitis; *β2MG* beta-2 microglobulin; *IgG4-RD* IgG4-related disease; *NA* not available; *NAG* N-acetyl-beta-glucosaminidase; *PBC* primary biliary cholangitis; *SLE* systemic lupus erythematosus;

The IgG subclass rates of the four groups are shown in Fig. 1. The IgG subclass rates had significant differences in IgG1 (*p* = 0.0362) and IgG4 (*p* < 0.01) between IgG4-RD and the other groups. There were no significant differences in percentages of IgG2 (*p* = 0.344) or IgG3 (*p* = 0.115) positive plasma cells between IgG4-RD and the other groups. In non-IgG4-RD groups, IgG1 was the highest subclass, with a median of 73.1% (mean 71.6%) in the autoimmune disease group (*p* = 0.157), 53.5% (mean 52.2%) in the AAV group (*p* = 0.706), and 68.0% (mean 66.1%) in the unknown disease group (*p* = 0.0327). In the IgG4-RD group, IgG subclass rate was high in both IgG1 and IgG4, with a median of 46.8% (mean 43.9%) for IgG1 and 44.4% (mean 45.2%) for IgG4, respectively. In comparison with IgG4-RD group, the rate of average IgG4-positive cells was significantly lower in the autoimmune disease group (median 1.1%, mean 4.1%, *p* < 0.01) and in the unknown disease group (median 7.8%, mean 13.6%, *p* < 0.01). AAV group showed a relatively higher median value of 18.5% of IgG4-positive cells (mean 26.6%), and there were no significant differences (*p* = 0.259). Representative images of IgG subclass immunostaining in IgG4-RD and non-IgG4-related interstitial nephritis are shown in Fig. 2. Complete quantitative data of IgG1-, IgG2-, IgG3-, and IgG4-positive plasma cells in all cases are shown in Supplementary Table 2.

**Fig. 3:**
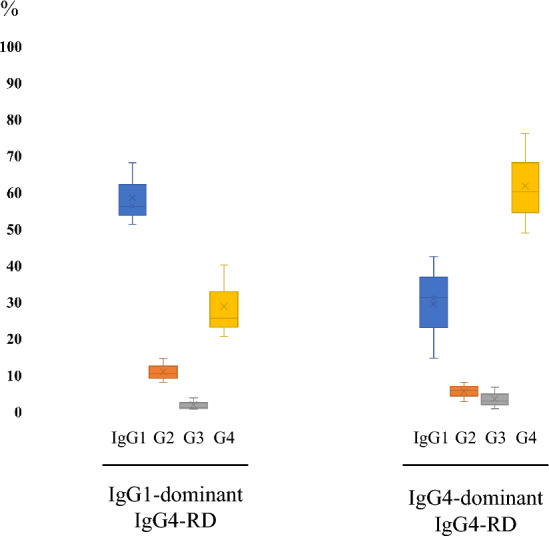
IgG subclass profiles of IgG4-related kidney disease. Average IgG subclass percentage of IgG1-dominant (left) and IgG4-dominant (right) IgG4-RD are shown in Box-and-Whisker plot. Blue, orange, gray, and yellow boxes indicate IgG1, IgG2, IgG3, and IgG4, respectively.

Further IgG subclass analysis revealed characteristic findings within specific subgroups. In the IgG4-RD group, three patients were IgG1-dominant and the other three were IgG4-dominant (Fig. 2). Comparison of the IgG subclass rate between these two groups is shown in Fig. 3. Except for the higher serum IgG4/IgG ratio in the IgG4-dominant group, there were no obvious differences in clinical or laboratory findings between these groups (Supplementary Table 3). IgG2-positive cells (median 8.0%, mean 8.2%) and IgG3-positive cells (median 2.0%, mean 2.6%) were notably low in IgG4-RD. IgG2-dominant subclass was detected in one case of PBC. In the AAV group, the IgG subclass rate was IgG1-dominant in seven cases and IgG4-dominant in two cases, which had no associations with ANCA antigen type. All of the pediatric cases were IgG1-dominant. Regarding the period from disease onset to the biopsy in non-IgG4-RD cases, IgG1-dominant immunophenotype was dominant (8/10) in cases that were diagnosed within 1 month.

## Discussion

In the present study, we investigated the IgG subclass of plasma cells in TIN associated with various etiologies. IgG subclass of TIN other than IgG4-RD had IgG1 predominance over the other subclasses. Other than in the AAV group, TIN of non-IgG4-RD also exhibited significantly lower percentages of IgG4 positivity compared with IgG4-RD. IgG2-dominant TIN was observed in only one case of PBC. There were no TIN cases of IgG3-dominant immunophenotype.

　In humans, IgG1 is the most abundant IgG subclass in the serum [10]. In the present study, IgG1 was the most prevalent IgG subclass of plasma cells in TIN. IgG1-producing plasma cells may therefore play a major role in immune responses in both peripheral blood and renal interstitium. IgG1 antibody binds efficiently to many immune cells, including FcγRI of monocytes, FcγRIIA and FcγRIIB of neutrophils, and FcγRIIIA of NK cells, activating the C1q classical pathway more efficiently than IgG4 [11]. Specific biological responses of IgG1 and IgG4 may account for the mechanistic insights into autoimmune pancreatitis, where IgG1 is highly pathogenic and IgG4 is speculated to attenuate the pathogenic effect of IgG1 [12]. Similarly, in the kidney, IgG1-dominant plasma cell infiltration may be associated with TIN tissue injury.

IgG subclass profiles may be useful in the differential diagnosis of TIN. The interstitial IgG4-positive plasma cell infiltration does not imply IgG4-related kidney disease, and some mimickers, including AAV-related interstitial nephritis, require differentiation [5]. Kawano et al. reported plasma cell infiltration of > 30/HPF in six of ten AAV cases, among which four cases had an IgG4/IgG ratio of > 40% [13]. Masuzawa et al. found very severe plasma cell infiltration in 3 out of 20 AAV cases, two of which had an IgG4/IgG ratio > 40% [14]. Diagnosis of IgG4-related kidney disease therefore requires consideration of multifactorial clinical and histological findings, such as the absence of serum ANCA, the degree of renal impairment and urinary abnormalities, and storiform fibrosis [9, 15]. Another potential diagnostic clue is that IgG1-dominant immunophenotypes of TIN may deserve consideration as a mimicker of IgG4-RD, irrespective of IgG4-positive plasma cell infiltration. In primary sclerosing cholangitis, patients have higher serum IgG1 levels than in IgG4-related cholangitis; this is further evidence that IgG subclass profiles may contribute to discrimination between IgG4-RD and mimickers [16].

TIN associated with IgG4-RD was characterized by a very high number of IgG4-positive plasma cells by definition. Further IgG subclass analysis divided IgG4-RD related-TIN into IgG1-dominant and IgG4-dominant subgroups. Yamaguchi et al. conducted immunohistological studies of IgG subclass in 16 cases of TIN associated with IgG4-RD, reporting one case of IgG1 predominant IgG4-RD [9]. In IgG4-RD involving the orbit, IgG2-positive plasma cells were more common than IgG4-positive plasma cells [17]. These results raise the possibility that the local immune network, not restricted to IgG4, may be involved in the heterogenic clinical and pathological profiles of IgG4-RD. Although the present study provided no significant clinical differences between IgG1-dominant and IgG4-dominant TIN associated with IgG4-RD, the small sample size means future investigations are warranted to elucidate the clinicopathological significance of IgG subclass distributions in IgG4-RD.

The IgG2 predominant subclass in PBC-associated TIN warrants further consideration. The predominant serum IgG subclasses in PBC have been reported to be IgG2 or IgG3 [18], which may be associated with abundant infiltration of IgG2-positive plasma cells in the renal tissue. In contrast, the serum IgG1 is significantly higher in patients with SLE and in patients with Sjogren’s syndrome than in healthy patients [19], potentially attributable to the IgG1-dominant positive cells in TIN associated with SLE and Sjogren’s syndrome. Compared with IgG1, IgG2 is less effective in activating the classical complement pathway via C1q binding [10]. Different profiles of IgG subclass may therefore be involved in the specific pathogenesis of TIN associated with autoimmune diseases.

IgG3 is produced earlier than other subclasses, with sequential class switching in the order of IgG3, IgG1, IgG2, and IgG4. Another unique property of IgG3 is the shorter half-life (7 days) compared with the 21 days of other subclasses [11]. These characteristic properties of IgG3 may be partly associated with early immune responses of TIN. Of three patients (cases 26, 31, and 35 shown in Supplementary Table 2) who had a relatively higher percentage of IgG3 (> 10%), two were diagnosed within 1 month after disease onset and the other was diagnosed relatively early, after 3 months. However, most cases of TIN within the early period had a lower proportion of IgG3 subclass, indicating a limited association between IgG3 and disease progression.

The present study has some limitations. Its retrospective nature shows that there is possible data collection bias. Also, the overall sample size is relatively small. In particular, the number of cases of TIN associated with IgG4-RD and PBC is substantially limited, and this is thought to affect the data interpretation in the subgroups. The incidence of TIN is relatively infrequent, ranging from 1 to 7% in children [20], 1 to 3% in adults [21], and it was 3.2% in a Japanese registry study [22]. The most common cause of TIN is the use of new drugs (32.4%) [22], TIN caused by autoimmune disease is mainly in Sjogren’s syndrome and SLE, while TIN due to PBC is quite rare (0.7%), and this is thought to inhibit data collection in large numbers [22]. IgG4-related kidney disease is also a rare chronic kidney disease (9.4%), and may be diagnosed by clinical symptoms, serum IgG and IgG4 levels, and biopsies of other organs [22]. Further investigation with a larger number of enrolled cases is required to elucidate the clinicopathological characteristics of TIN due to both PBC and IgG4-RD. Additionally, unknown disease group is 21 cases, constituting a large proportion in this study. The lack of specific disease background in this major group is another limitation to interpret the significance of IgG1-dominant immunophenotype. The causes of TIN are highly heterogeneous and the pathogenesis should not be judged solely on the basis of IgG subclass results, but need to consider comprehensively glomerular, tubulointerstitial and vascular lesions, as well as clinical findings. In terms of the diagnostic procedure, we acknowledge that IgG subclass analysis provides complementary information, not diagnostic results by itself.

In summary, the present study revealed IgG1-dominant immune profiles of TIN unrelated to IgG4-RD. IgG subclass profile may add some diagnostic merit of TIN. This needs to be confirmed by the studies enrolled with larger numbers of TIN cases. The relatively higher level of IgG4-positive cells in the AAV group may reflect partially overlapping immune responses of IgG4-RD. Further investigation is required to elucidate the clinicopathological differences between IgG1-dominant and IgG4-dominant groups in IgG4-RD.

### Supplementary Information

Below is the link to the electronic supplementary material.

Supplementary file1 (DOCX 59 KB)

## Data Availability

The authors confirm that the data supporting the findings of this study are available within the article and its supplementary materials.

## Abbreviations

AAV: Antineutrophil cytoplasmic antibody (ANCA)-associated vasculitis
ANCA: Antineutrophil cytoplasmic antibody
C3: Complement component 3
Cr: Creatinine
e-GFR: Estimated glomerular filtration rate
IgG4-RD: IgG4-related disease
MN: Membranous nephropathy
NAG: Urinary *N*-acetyl-beta-glucosaminidase
SLE: Systemic lupus erythematosus
TIN: Tubulointerstitial nephritis
β2MG: Urinary beta-2 microglobulin
