# Supplementary material for: Immunohistological analysis reveals IgG1-dominant immunophenotype of tubulointerstitial nephritis unassociated with IgG4-related diseases
Source: Int Urol Nephrol. 2024 Feb 21;56(7):2363–9. doi: 10.1007/s11255-024-03966-1 (PMC11189997; doi:10.1007/s11255-024-03966-1)
Supplement: Supplementary file 1 — Supplementary file1 (DOCX 59 KB) [file 11255_2024_3966_MOESM1_ESM.docx]

**Supplementary table1:** Main clinical data for all tubulointerstitial nephritis cases

|  | Clinical | | | | Serum/Urinary test | | | | | | | | |
| --- | --- | --- | --- | --- | --- | --- | --- | --- | --- | --- | --- | --- | --- |
| Case | Age | Sex | Cause | Period ^1^ | IgG (mg/dl) | IgG4 (mg/dl) | Cr (mg/dl) | eGFR (ml/min/1.73m^2^) | C3 (mg/dl) | Hematuria  (−/±/1+/2+/3+) | Proteinuria  (−/±/1+/2+/3+/4+) | β2MG (μg/L) | NAG (IU/L) |
| 1 | 50 | M | IgG4 | 1 y 4 m | 2048 | 682 | 1.12 | 56.1 | 105 | - | - | 994 | 2.9 |
| 2 | 59 | M | IgG4 | 3 m | 4703 | 3120 | 1.54 | 38 | 62 | - | ± | 8088 | 8.7 |
| 3 | 63 | M | IgG4 | 2 m | 3558 | 953 | 0.92 | 65 | NA | - | ± | 40 | 11.4 |
| 4 | 74 | F | IgG4 | 2 y | 4936 | 722 | 1.4 | 28.8 | 40 | 2+ | 1+ | 7335 | 4.4 |
| 5 | 76 | M | IgG4 | 9 m | 2449 | 855 | 1.11 | 49.9 | 47 | 1+ | 3+ | 445 | 51.5 |
| 6 | 81 | M | IgG4 | 2 m | 3336 | 288 | 1.45 | 37 | 50 | ± | 1+ | 39667 | 31.3 |
| 7 | 14 | M | Sjogren’s syndrome | 2–3 m | NA | NA | NA | NA | NA | NA | NA | NA | NA |
| 8 | 39 | F | Sjogren’s syndrome | 2 y | 2287 | 29.5 | 1.22 | 40 | 117 | - | - | 6287 | 4.5 |
| 9 | 40 | F | Sjogren’s syndrome | Unk | 2060 | 17.2 | 0.65 | 79.7 | 101 | 1+ | ± | 18176 | 11 |
| 10 | 49 | F | Sjogren’s syndrome | 14 y | 1576 | NA | 1.05 | 44.5 | 99 | 1+ | 1+ | 21500 | 7.9 |
| 11 | 68 | F | Sjogren’s syndrome | Unk | 6352 | 12.6 | 1.24 | 34 | 69 | - | ± | NA | 30.7 |
| 12 | 51 | M | PBC | 3 m | 3117 | 63.5 | 1.75 | 34 | 112 | - | ± | 28267 | 4.7 |
| 13 | 70 | F | PBC | 2 y | 1016 | 15.8 | 1.42 | 29 | NA | ± | 2+ | 43060 | 5.4 |
| 14 | 46 | F | SLE | Unk | 1795 | NA | 1.04 | 45.8 | 24 | 2+ | 2+ | 9861 | 3.6 |
| 15 | 46 | M | AAV（MPO） | 1.5 m | 1246 | NA | 0.95 | 68.4 | 45.3 | 2+ | 3+ | 140 | 17.7 |
| 16 | 52 | F | AAV（MPO） | 3 m | 1424 | NA | 0.66 | 72.7 | 118 | 3+ | 3+ | 267 | 14.4 |
| 17 | 59 | F | AAV（MPO） | 1.5 m | 1220 | NA | 2.9 | 13.9 | 126 | 3+ | 3+ | 565 | 31.7 |
| 18 | 67 | F | AAV（MPO） | 1 w | 1797 | NA | 0.96 | 44.8 | 126 | 1+ | 3+ | 3050 | 0.27 |
| 19 | 72 | M | AAV（MPO） | 1 m | 1035 | NA | 0.95 | 60.1 | 82 | 2+ | 3+ | 1383 | 21.8 |
| 20 | 75 | M | AAV（MPO） | 3 m | 1551 | NA | 3.45 | 14 | NA | 1+ | 2+ | 23641 | 16.5 |
| 21 | 76 | F | AAV（MPO） | 2 w | 1203 | NA | 4.41 | 8.2 | 110 | 1+ | 3+ | 569 | 18.3 |
| 22 | 46 | M | AAV（PR3） | 3 m | 480 | NA | 4.15 | 13.6 | 29.5 | 3+ | 3+ | 10235 | 24.4 |
| 23 | 62 | F | AAV（PR3） | 3 m | 2291 | NA | 1.34 | 32 | 82 | 1+ | 2+ | 3392 | 7.9 |
| 24 | 13 | M | Unk | 1 m | 2012 | 10 | 1.63 | 42.2 | 115 | - | ± | 22073 | 15.8 |
| 25 | 15 | M | Unk | 2 y | 1306 | NA | 1.96 | 29.4 | 153.4 | - | - | 10903 | 2.1 |
| 26 | 36 | M | Unk | 1 m | 1248 | NA | 3.7 | 17 | 137 | ± | 1+ | 70982 | 14.4 |
| 28 | 40 | M | Unk | 3 m | 383 | NA | 2.03 | 31 | 111 | ± | 4+ | 850 | 13 |
| 27 | 42 | F | Unk | 2 y | 1506 | 33.8 | 1.59 | 30 | 96 | - | - | 13232 | 3.8 |
| 29 | 45 | M | Unk | 2 m | 1710 | NA | 1.95 | 35 | NA | ± | 2+ | 96070 | 10 |
| 30 | 48 | M | Unk | Unk | 1588 | NA | 10.98 | 5 | 95 | 1+ | 1+ | 10575 | 15.9 |
| 31 | 54 | M | Unk | 1 w | 1670 | NA | 16.94 | 2 | 128 | 3+ | 3+ | 5176 | 19 |
| 32 | 55 | F | Unk | 2 m | 1300 | 37.7 | 3.72 | 11 | 118 | - | 1+ | 27621 | NA |
| 33 | 58 | M | Unk | 9 y | 2220 | NA | 2.56 | 21.6 | 137 | - | 1+ | NA | NA |
| 34 | 62 | F | Unk | 3 m | 1590 | 61.2 | 1.16 | 37 | 79 | ± | - | 26770 | 10.2 |
| 35 | 66 | M | Unk | 3 m | 1830 | 63 | 2.97 | 17.7 | 96 | 1+ | 2+ | 75700 | 13.7 |
| 36 | 66 | F | Unk | 6 m | 1786 | 35.2 | 1.67 | 25 | 106 | 1+ | 1+ | 51793 | 15.2 |
| 37 | 66 | F | Unk | 4 m | 585 | 7.4 | 1.07 | 40 | 93 | 1+ | 1+ | 39948 | 12.8 |
| 38 | 67 | F | Unk | 1 m | 2453 | 1090 | 5.84 | 6 | 98 | 1+ | 1+ | 16435 | 20.2 |
| 39 | 68 | M | Unk | 2 w | 1900 | NA | 2.23 | 24 | 96 | 2+ | 1+ | 13652 | 41.9 |
| 40 | 70 | F | Unk | 50 d | 1790 | NA | 1.65 | 24.5 | 142 | 3+ | ± | 4168 | 10.1 |
| 41 | 72 | F | Unk | 2 m | 1844 | NA | 2.19 | 18 | 167 | 2+ | 2+ | 6118 | 19.6 |
| 42 | 75 | M | Unk | 1 m | 1618 | NA | 5.1 | 9 | 99 | 3+ | 1+ | 29576 | 11.2 |
| 43 | 76 | M | Unk | 1 m | 2647 | NA | 4.83 | 10 | 111 | 3+ | 1+ | 46170 | 31.8 |
| 44 | 78 | M | Unk | 1 m | 1664 | NA | 3.23 | 15 | 129 | 1+ | 1+ | 193 | 43.2 |

Period ^1^ indicates the “Period from patient’s symptom until biopsy”（w, week; m, month; y, year）

Abbreviation: AAV, ANCA-associated vasculitis; β2MG, beta-2 microglobulin; F, female; IgG4-RD, IgG4-related disease; M, male; MPO, myeloperoxidase; NA, not available; NAG, N-acetyl-beta-glucosaminidase; PR3, proteinase 3; SLE, systemic lupus erythematosus; Unk: Unknown

**Supplementary table2:** IgG subclass data for all tubulointerstitial nephritis cases

|  |  |  |  |  | Count average（/x400） | | | | Average（%） | | | |
| --- | --- | --- | --- | --- | --- | --- | --- | --- | --- | --- | --- | --- |
| Case | Age | Sex | Cause | Period ^1^ | IgG1 | IgG2 | IgG3 | IgG4 | IgG1 | IgG2 | IgG3 | IgG4 |
| 1 | 50 | M | IgG4 | 1 y 4 m | 92.7 | 17.3 | 8.7 | 179.0 | 31.1 | 5.8 | 2.9 | 60.1 |
| 2 | 59 | M | IgG4 | 3 m | 66.3 | 12.0 | 30.3 | 344.0 | 14.7 | 2.7 | 6.7 | 76.0 |
| 3 | 63 | M | IgG4 | 2 m | 79.3 | 12.3 | 1.0 | 62.0 | 51.3 | 8.0 | 0.6 | 40.1 |
| 4 | 74 | F | IgG4 | 2 y | 264.3 | 68.7 | 17.7 | 120.0 | 56.2 | 14.6 | 3.8 | 25.5 |
| 5 | 76 | M | IgG4 | 9 m | 202.3 | 38.3 | 3.7 | 233.0 | 42.4 | 8.0 | 0.8 | 48.8 |
| 6 | 81 | M | IgG4 | 2 m | 61.7 | 9.3 | 1.0 | 18.7 | 68.0 | 10.3 | 1.1 | 20.6 |
| 7 | 14 | M | Sjogren’s syndrome | 2–3 m | 27.3 | 4.3 | 0.0 | 0.7 | 84.5 | 13.4 | 0.0 | 2.1 |
| 8 | 39 | F | Sjogren’s syndrome | 2 y | 87.3 | 3.7 | 4.0 | 1.7 | 90.3 | 3.8 | 4.1 | 1.7 |
| 9 | 40 | F | Sjogren’s syndrome | Unk | 101.0 | 6.7 | 0.0 | 0.3 | 93.5 | 6.2 | 0.0 | 0.3 |
| 10 | 49 | F | Sjogren’s syndrome | 14 y | 17.7 | 13.7 | 1.7 | 0.3 | 53.0 | 41.0 | 5.0 | 1.0 |
| 11 | 68 | F | Sjogren’s syndrome | Unk | 111.0 | 1.3 | 0.3 | 0.3 | 98.2 | 1.2 | 0.3 | 0.3 |
| 12 | 51 | M | PBC | 3 m | 53.3 | 65.3 | 12.3 | 1.7 | 40.2 | 49.2 | 9.3 | 1.3 |
| 13 | 70 | F | PBC | 2 y | 36.3 | 19.3 | 3.0 | 0.3 | 61.6 | 32.8 | 5.1 | 0.6 |
| 14 | 46 | F | SLE | Unk | 92.7 | 34.7 | 8.0 | 45.7 | 51.2 | 19.2 | 4.4 | 25.2 |
| 15 | 46 | M | AAV（MPO） | 1.5 m | 6.0 | 5.0 | 0.0 | 4.0 | 40.0 | 33.3 | 0.0 | 26.7 |
| 16 | 52 | F | AAV（MPO） | 3 m | 17.0 | 9.3 | 0.0 | 2.3 | 59.3 | 32.6 | 0.0 | 8.1 |
| 17 | 59 | F | AAV（MPO） | 1.5 m | 7.7 | 2.7 | 0.0 | 4.0 | 53.5 | 18.6 | 0.0 | 27.9 |
| 18 | 67 | F | AAV（MPO） | 1 w | 116.3 | 43.3 | 0.0 | 23.7 | 63.5 | 23.6 | 0.0 | 12.9 |
| 19 | 72 | M | AAV（MPO） | 1 m | 38.7 | 10.3 | 0.3 | 1.7 | 75.8 | 20.3 | 0.7 | 3.3 |
| 20 | 75 | M | AAV（MPO） | 3 m | 40.0 | 27.3 | 0.0 | 15.3 | 48.4 | 33.1 | 0.0 | 18.5 |
| 21 | 76 | F | AAV（MPO） | 2 w | 14.0 | 1.7 | 0.0 | 26.7 | 33.1 | 3.9 | 0.0 | 63.0 |
| 22 | 46 | M | AAV（PR3） | 3 m | 85.3 | 19.7 | 0.7 | 11.7 | 72.7 | 16.8 | 0.6 | 9.9 |
| 23 | 62 | F | AAV（PR3） | 3 m | 3.3 | 0.0 | 1.0 | 9.7 | 23.8 | 0.0 | 7.1 | 69.0 |
| 24 | 13 | M | Unk | 1 m | 100.3 | 5.0 | 0.0 | 0.3 | 95.0 | 4.7 | 0.0 | 0.3 |
| 25 | 15 | M | Unk | 2 y | 26.7 | 8.0 | 0.0 | 3.7 | 69.6 | 20.9 | 0.0 | 9.6 |
| 26 | 36 | M | Unk | 1 m | 13.7 | 3.3 | 2.3 | 0.3 | 69.5 | 16.9 | 11.9 | 1.7 |
| 27 | 40 | M | Unk | 3 m | 5.3 | 2.0 | 0.0 | 1.7 | 59.3 | 22.2 | 0.0 | 18.5 |
| 28 | 42 | F | Unk | 2 y | 43.0 | 12.3 | 1.0 | 2.7 | 72.9 | 20.9 | 1.7 | 4.5 |
| 29 | 45 | M | Unk | 2 m | 64.3 | 1.3 | 0.0 | 0.7 | 97.0 | 2.0 | 0.0 | 1.0 |
| 30 | 48 | M | Unk | Unk | 23.7 | 11.0 | 1.3 | 4.0 | 59.2 | 27.5 | 3.3 | 10.0 |
| 31 | 54 | M | Unk | 1 w | 16.7 | 0.3 | 5.0 | 1.0 | 72.5 | 1.4 | 21.7 | 4.3 |
| 32 | 55 | F | Unk | 2 m | 19.3 | 4.3 | 0.0 | 2.0 | 75.3 | 16.9 | 0.0 | 7.8 |
| 33 | 58 | M | Unk | 9 y | 32.0 | 3.3 | 1.7 | 17.3 | 58.9 | 6.1 | 3.1 | 31.9 |
| 34 | 62 | F | Unk | 3 m | 44.0 | 7.0 | 0.7 | 4.7 | 78.1 | 12.4 | 1.2 | 8.3 |
| 35 | 66 | M | Unk | 3 m | 26.7 | 20.0 | 9.7 | 3.0 | 44.9 | 33.7 | 16.3 | 5.1 |
| 36 | 66 | F | Unk | 6 m | 42.7 | 38.3 | 3.7 | 10.3 | 44.9 | 40.4 | 3.9 | 10.9 |
| 37 | 66 | F | Unk | 4 m | 14.3 | 0.0 | 0.0 | 0.0 | 100.0 | 0.0 | 0.0 | 0.0 |
| 38 | 67 | F | Unk | 1 m | 0.3 | 8.0 | 0.0 | 39.3 | 0.7 | 16.8 | 0.0 | 82.5 |
| 39 | 68 | M | Unk | 2 w | 118.0 | 62.7 | 0.3 | 0.7 | 65.0 | 34.5 | 0.2 | 0.4 |
| 40 | 70 | F | Unk | 50 d | 57.3 | 22.0 | 4.3 | 6.7 | 63.5 | 24.4 | 4.8 | 7.4 |
| 41 | 72 | F | Unk | 2 m | 17.0 | 6.0 | 0.0 | 2.0 | 68.0 | 24.0 | 0.0 | 8.0 |
| 42 | 75 | M | Unk | 1 m | 26.7 | 4.0 | 0.3 | 17.0 | 55.6 | 8.3 | 0.7 | 35.4 |
| 43 | 76 | M | Unk | 1 m | 104.7 | 18.7 | 1.0 | 10.0 | 77.9 | 13.9 | 0.7 | 7.4 |
| 44 | 78 | M | Unk | 1 m | 131.7 | 10.7 | 4.0 | 67.7 | 61.5 | 5.0 | 1.9 | 31.6 |

Period ^1^ indicates the time from patient’s symptom until biopsy（w, week; m, month; y, year）.

Abbreviation: AAV, ANCA-associated vasculitis; F, female; IgG4-RD, IgG4-related disease; M, male; MPO, myeloperoxidase; PR3, proteinase 3; SLE, systemic lupus erythematosus; Unk: Unknown

**Supplementary table 3:** Relationship of clinical data and laboratory values against two different dominant types of IgG4-related disease.

|  | IgG4-RD（IgG1-dominant） | | | IgG4-RD（IgG4-dominant） | | |
| --- | --- | --- | --- | --- | --- | --- |
|  | （n = 3） | | | （n = 3） | | |
| Patient number | 3 | 4 | 6 | 1 | 2 | 5 |
| Age | 63 | 74 | 81 | 50 | 59 | 76 |
| Sex | M | F | M | M | M | M |
| Period（month）^1^ | 2 | 24 | 2 | 16 | 3 | 9 |
| IgG（mg/dl） | 3558 | 4936 | 3336 | 2048 | 4703 | 2449 |
| IgG4（mg/dl） | 953 | 722 | 288 | 682 | 3120 | 855 |
| IgG4/IgG rate | 0.27 | 0.15 | 0.09 | 0.33 | 0.66 | 0.35 |
| Cr（mg/dl） | 0.92 | 1.4 | 1.45 | 1.12 | 1.54 | 1.11 |
| eGFR（ml/min/1.73 m^2^） | 65 | 28.8 | 37 | 56.1 | 38 | 49.9 |
| C3（mg/dl） | no data | 40 | 50 | 105 | 62 | 47 |
| Hematuria（−/±/1+/2+/3+） | - | 2+ | ± | - | - | 1+ |
| Proteinuria（−/±/1+/2+/3+） | ± | 1+ | 1+ | - | ± | 3+ |
| β2MG（μg/L） | 40 | 7335 | 39667 | 994 | 8088 | 445 |
| NAG（μg/L） | 11.4 | 4.4 | 31.3 | 2.9 | 8.7 | 51.5 |
| IgG1（%） | 51.3 | 56.2 | 68.0 | 31.1 | 14.7 | 42.4 |
| IgG2（%） | 8.0 | 14.6 | 10.3 | 5.8 | 2.7 | 8.0 |
| IgG3（%） | 0.6 | 3.8 | 1.1 | 2.9 | 6.7 | 0.8 |
| IgG4（%） | 40.1 | 25.5 | 20.6 | 60.1 | 76.0 | 48.8 |

Period ^1^ indicates the time from patient’s symptom until biopsy.

Abbreviation: IgG4-RD, IgG4-related disease;
